# Determination of trace levels of organic fining agents in wines: Latest and relevant findings

**DOI:** 10.3389/fchem.2022.944021

**Published:** 2022-08-05

**Authors:** David Bongiorno, Giuseppe Avellone, Anna Napoli, Fabio Mazzotti, Daniela Piazzese, Valentina Censi, Serena Indelicato

**Affiliations:** ^1^ Dipartimento Scienze e Tecnologie Biologiche Chimiche e Farmaceutiche (STEBICEF)—Università degli Studi di Palermo—via Archirafi *,* Palermo *,* Italy; ^2^ Dipartimento di Chimica e Tecnologie Chimiche, Università della Calabria Arcavacata di Rende, Calabria, Italy; ^3^ Dipartmento di Scienze della Terra e del Mare—Università degli Studi di Palermo—via Archirafi, Palermo, Italy

**Keywords:** fining agents, wine, mass spectrometry, ELISA: enzyme-linked immunosorbent assay, biosensors, allergens

## Abstract

The production of red wine plays a key role in the local and international economies of several nations. During the winemaking process, to clarify the final product, before bottling, and to remove undesired substances (proteins, phenols, and tannins), fining agents are commonly added to wines. These substances have different origins (animal and vegetable proteins or mineral compounds), and they show a potential risk for the health of allergic subjects. For these reasons, the residues of fining agents, constituted by exogenous proteins based on gluten, egg, and milk proteins, should not be present in the final product and their trace residues should be quantified with accuracy. In the last decade, several analytical approaches have been developed for their quantitative determination using different sample treatment protocols and analytical techniques. These methods are based on liquid chromatography coupled with mass spectrometry or enzyme-linked immunosorbent assays (ELISAs). Recently, biosensors have been proposed as a potential alternative to immunoassay approaches, allowing rapid, cheap, and simple multi-residue detection. This short review aimed to report the most recent and relevant findings in the field.

## Introduction

In winemaking, the term “fining” indicates a clarification step that implies the addition of substances (fining agents) deemed to reduce or remove some undesirable compounds that could give rise to unpleasing precipitates. The fining process also modifies the wines’ organoleptic characteristics, reduces astringency, and improves color and flavor. Fining agents are removed according to good manufacturing practices, but these substances are considered “processing aids” and should not be present in the final product. According to the FDA (2013), fining agents could not be reported on the wine label, if removed during the winemaking. But the permanence of “exogenous” compounds in the final products must be evaluated, taking into account their origin (animal or vegetable proteins, mineral compounds) and mostly their potential consequence on the health of the consumer. As aforesaid, the arsenal of fining agents at disposal of the winemaker is quite ample, but the best results often require the adoption of synergistic action of many clarifiers. Among the non-proteic organic compounds used in beverage fining, polyvinylpolypyrrolidone (PVPP), reported as generally recognized as safe (GRAS) and approved for many uses by the FDA, is particularly useful as it is capable of removing flavans and phenols (Ronnau et al., 2000; Adachi et al., 2003; Yoshida et al., 2008). PVPP, by removing phenols, also lowers the bitterness and prevents oxidative browning in white wines. As a collateral effect, PVPP depletes resveratrol (Threlfall et al., 1999) and quercetin (Laborde et al., 2006) commonly associated with the healthy effect of moderate wine consumption (Castellari et al., 1998). The adoption of proteic clarifiers, while preserving most of the healthy substances, can represent a risk for hypersensitive consumers, even if the literature seems to indicate the inconsistency of an allergological risk. Indeed, while proteinaceous fining agents are used to obtain the tannin precipitation, the formation of soluble complexes between tannins and proteins takes place. These complexes can remain in the solution even in correctly processed fined wines. According to Maury et al. (2019), in a study involving the preparation of a model wine, fined with radio-labeled proteins (gelatin or wheat gluten protein), their residue amounted from 24% to 58% of the initial fining proteins added. Finally, the use of animal proteins could also entail ethical or religious concerns, such as vegetarianism or veganism, or religious faith. For this reason, since 31 May 2009, the EU Directive 2007/68/EC established that all wines must declare on the label if proteic allergens have been used during wine production. Similar regulations on the use of eggs, milk, and fish derivatives (isinglass) as clarifiers of wines and stabilizers have also been enacted in other countries such as the United States, New Zealand, and Australia. However, showing these warnings on the label could reduce the attractiveness of the product and potentially damage the perception of its quality.

## Classification of protein-based fining agents used in winemaking

Several fining agents are of proteic nature (Marangon et al., 2019), and we can find animal or plant proteins. The former are frequently obtained from collagen (i.e., bovine and porcine gelatin) (Hrazdina et al., 1969; Oberholster et al., 2013), fish gelatin and isinglass (Sanborn et al., 2010), milk (caseinates) (Weber et al., 2009), and white egg (ovalbumin). Among plant proteins, we consider those derived from cereals (Simonato et al., 2009; Iturmendi et al., 2010; Simonato et al., 2011; Simonato et al., 2013), legumes (Granato et al., 2018), grape seeds, potatoes (Gambuti et al., 2012; Gambuti et al., 2016), and seaweeds (Noriega-Domínguez et al., 2010).

Since 2000, several food production protocols have been modified due to the spread of bovine spongiform encephalopathy. Winemaking was no exception, and some of the fining agents of animal origin (bovine proteins) have been abandoned. Consequently, among clarifying agents, porcine gelatin remained the most commonly used protein (Sarni-Manchado et al., 1999; Maury et al., 2001; Smith et al., 2015).

The gelatin (from pig and fish) at low pH gives rise to colloidal particles that, being positively charged, interact with negatively charged particles, originating sediments. Among the fish gelatins, isinglass is very pure gelatin, prepared from the air bladders or, more recently, from other fish tissues (Rizzi et al., 2016).

On the other hand, caseins, which are phosphoproteins obtained from milk, contain sequences of hydrophilic and hydrophobic amino acids, which confer an amphiphilic nature to these fining agents. At the pH of the wine, caseins in association with sodium or potassium form insoluble micelles, which leads to coagulation and sedimentation of the interacting substances (Weber et al., 2007a). In addition, caseins lead to the formation of insoluble complexes with phenolic compounds from grapes and allow removing the excess tannin in over-oaked white wines (Weber et al., 2009; Cosme et al., 2012).

Egg proteins are largely used as clarifying agents due to their ability to bind and reduce tannin content (Cosme et al., 2007; Rizzi et al., 2016). As fish gelatins, they are positively charged and form aggregates with the negatively charged particles of wines. The lysozyme, another egg protein, shows antimicrobial activity against Gram-positive bacteria and, thus, is used as a stabilizer for better control of the fermentation process and against wine spoilage (Weber et al., 2007a). As an alternative to animal proteins, those derived from legumes (pea, lentils, and soy), wheat gluten, and oenological yeast protein extracts (Gaspar et al., 2019) can be used for fining purposes (Marangon et al., 2019).

This notwithstanding, some of them still represent a potential risk to the health of allergic subjects, and therefore have to be traced in the final product. Simonato et al. (2009; 2013) reported that Patatin P (Gambuti et al., 2012) and maize zeins could be used as fining agents. Patatin P, which belongs to glycoproteins, is obtained from a potato aqueous by-product, and it could represent a vegetable-based clarifying agent, with the same properties of animal protein used in winemaking. Maize zeins, extracted from the “corn gluten,” plays a good role as a processing aid in winemaking and has the advantage of not requiring a label declaration (European Commission. Directive 2003/89/EC; European Commission. Directive 2007/68/EC).

Cosme et al. (2012) and Kang et al. (2018) investigated a possible application of rice proteins as clarifying agents with properties similar to caseinates for white and rosè wines and gelatin for red wines.

More recently, grape seed proteins have been tested as clarifiers (Vincenzi et al., 2013; Gazzola et al., 2017). These substances overcome the allergological risk related to the use of exogenous proteins during winemaking because the grape seed proteins are considered endogenous components of wine. Finally, Pino Ramos et al. (2022) have studied the fining ability of quinoa proteins as an alternative to animal proteins (i.e., gelatin).

## Analytical approaches for identifying exogenous protein in wines

The lack of stringent regulation related to the use of fining agents for the winemaking process and the possibility of adopting proteic clarifiers determines the possible persistence of exogenous proteins in the final product. Therefore, to evaluate their presence and to overcome allergological or food ethics problems, sensitive and accurate detection methods have been developed. Indeed, in the last decade, immunochemical assays, mass spectrometry (MS)-based analytical determinations, and biosensors have been applied to inform allergic consumers about the potential presence of allergens.

### Immunochemical assays

The enzyme-linked immunosorbent test (ELISA) represents a useful tool for the detection of exogenous protein residues in wine. It is characterized by easy execution, low cost, rapidity, and good sensitivity. ELISA tests are capable of detecting contamination of egg white, casein, and gluten, and are also useful for identifying and quantifying gelatins. The drawbacks in ELISAs come from the complexity of matrix composition, which can inhibit the immuno-enzymatic reaction (Koestel et al., 2016).

In addition, due to the absorption on the membrane surfaces, and the low pH value of wine and the content of tannins, the ability to interact with proteins in some epitopes could change, causing alterations, impairing sensitivity, or leading to false negatives or to partial responses (Kaul et al., 2007).

Weber et al. (2007a) reported LOD values, in the order of 0.005 μg/ml for the fish gelatin and lysozyme assays, that were complying with the Organisation Internationale de la Vigne et du Vin (OIV, Paris, France requests (values ≤ 0.25 μg/ml)), but not satisfying for other clarifying agents. Deckwart et al. (2014), using an indirect ELISA approach for investigating wine samples, obtained LOD values of caseins in the order of 0.2 μg/ml for red wines, while for white wines, the values ranged from 0.01 μg/ml to 0.1 μg/ml. Different sample pretreatments, based on immunochemical assays, have been proposed to determine various types of exogenous proteins in several commercial wines. Weber et al. (2007b) analyzed four white wines made in Germany, and after a dilution in the ratio of 1:10 in phosphate-buffered saline solution, it was possible to detect lysozyme residues in all of them. Egg albumin was found in just one wine that was refined with a high dosage of dried egg white (20 g/hl). Rolland et al. (2008) tested 153 commercial Australian wines, using a different sample pretreatment for white or red wine. The former was dialyzed (3.5 kDa cutoff) in SnakeSkin pleated dialysis tubing; the latter was simply diluted 1:4 in ethanol; no residual milk or egg proteins were found. Restani et al. (2012) analyzed 63 commercial wines (all filtered through membranes having a pore size of 1 µm) and 16 experimental wines treated with milk proteins (filtered through membranes having a pore size of 3 µm) using both ELISA and immunoblotting techniques, incubating the membranes with specific anti-caseinate antibodies. The authors did not find any detectable protein residue in the wines. Uberti et al. (2014), adopting the same pretreatment approach as Restani et al. (2012), investigated 78 commercial red wines from Australia, New Zealand, and Europe, reporting the oenological practices adopted and the corresponding level of egg white protein residues. The authors did not find egg proteins (LOD 0.0564 μg/ml), despite the wide range of doses (3–10 g/hl) used for refining. Simonato et al. (2011) precipitated wine proteins by means of the KDS method, originally proposed by Vincenzi et al. (2005), which involves protein complexation with dodecyl sulfate, followed by a precipitation step of complexes as potassium salts. They reported that wheat protein residues were detectable only in wines treated with massive gluten amounts (>50 g/hl), far higher than those usually employed in winemaking protocols. Zeleňáková et al. (2021) reported the identification of cow milk allergen in 17 wine samples (vintage 2014–2017) originating from Slovenia. The LOD was 0.24 μg/ml and the LOQ was 1.30 μg/ml. The sample treatment was very quick and easy: the extraction buffer solution (10 ml) was added to 1.00 ml of wine; the sample, maintained under continuous shaking for 5 min, was centrifuged; and the supernatant was sampled for the ELISA test. The findings showed the casein concentrations ranged from 1.634 to 16.715 μg/ml for white wines and from 21.473 to 67.22 μg/ml for red ones.

### MS-based methods

As reported elsewhere (Monaci et al., 2010; Maury et al., 2019), the drawbacks of immunochemical assays are mostly related to the formation of soluble protein–tannin complexes, which are not removed by filtration and potentially not recognized by ELISA antibodies. MS-based approaches are far less selective but extremely sensitive, and make it possible to determine an ample collection of molecules ranging from contaminants, antioxidants, polymers, surfactants, and proteins (Indelicato et al., 2016a; Di Donna et al., 2017; Aiello et al., 2020; Bongiorno et al., 2021). MS allows the detection at trace levels of fining agent residues while their identification is independent of the structure of allergens (Kaul et al., 2007; Kirsch et al., 2009; Picariello et al., 2011).

Tolin et al. (2012a), Tolin et al. (2012b), based on the same analytical approach as Simonato et al. (2011), scrutinized several commercial wines to ascertain the presence of animal protein residues. The nano-liquid chromatography (LC) tandem mass spectrometry (MS/MS) analytical strategy allowed the determination of ovalbumin and caseins at levels of about 100 ng/L and 60 ng/L of wine, respectively. Using this approach, the authors overcome the limitations of classical immunological methods, gaining sensitivity and eliminating the problems affecting immunoassays that rely on the labile tertiary structure of proteins (Kaul et al., 2007) for protein recognition and reactivity.

Monaci et al. (2013) developed an interesting approach based on isotopically labeled peptides of ovalbumin and *α* S1–casein to determine egg proteins and caseinates in white wine samples. This approach allowed to fairly estimate and take into account tryptic digestion yields in the analytical procedure. Good LODs were also achieved, ranging between 0.4 and 1.1 μg/ml (depending on the peptide considered as a marker of the fining agent). In order to reduce the analysis costs, Losito et al. (2013) proposed an approach for the determination of caseinate residues in refined white wines using LC coupled with a 3D ion trap mass spectrometer. The optimization of an appropriate wine volume and a protein extraction/digestion protocol, followed by MS/MS analysis performed on an ion trap instrument, provided a good sensitivity (LOD lower than 0.25 μg/ml) complying with the request of OIV, which represents the reference value in the current European legislation.

Another research aspect being pursued is the continuous refinement of the wine sample pretreatment. With this goal, Mattarozzi et al. (2014) proposed an LC-MS/MS method for the simultaneous determination of α-, β-casein, and ovalbumin in commercial red wine after a thorough evaluation of different sample treatments. According to the authors, among the most widely adopted strategies involving the use of denaturing agents, protein precipitation, cutoff filters, or size exclusion purification cartridges, the size exclusion-based procedure provided the best performance in terms of both accuracy (recovery) and precision. The validated LC-MS/MS method has proven sensitive enough to identify and quantify allergens in red wine protein extracts at trace levels, with LODs and LOQs values ranging from 0.01 to 0.8 μg/ml and from 0.03 to 2 μg/ml, respectively. De Angelis et al. (2017) succeeded in the upgrade of an analytical workflow based on the use of pre-enrichment columns. The sample clean-up was based on either size exclusion columns (SEC) or cut-off filters (UF), followed by selective peptide enrichment on a 1-cm-long C-18 trap column. It was found that the SEC-based procedure provided faster analyses, but the lengthier UF was the most sensitive approach, resulting in LODs and LOQs of 0.036 and 0.12 μg/ml for egg and 0.050 and 0.17 μg/ml for milk, respectively. Pilolli et al. (2014) evaluated two MS-based approaches: LC- high resolution (HR) MS and LC-MS/MS. These two approaches usually have different applications (Di Stefano et al., 2012; Indelicato et al., 2016b), but have proven to be equally well-suited for quantification and screening purposes. As can be foreseen, the main advantage of HR-MS lies in the open possibility of retrospective analysis. On the other hand, the MS/MS method was more sensitive to analytes and less sensitive to matrix. The same authors developed a new approach based on the integration of mass spectrometry and immunoassay (MSIA) (Pilolli et al., 2017) to detect egg allergens. Polyclonal antibodies raised against native ovalbumin were immobilized onto MSIA-customized disposable tips. The protocol implied a 1:4 dilution of the wine sample, an automated purification/enrichment step on MSIA, followed by tryptic digestion and LC-MS/MS determination. LOD and LOQ values obtained were 0.01 and 0.03 μg/ml, respectively.

The development of LC-MS/MS methods for the detection of caseins, albumin, and lysozyme in wines brought more refined approaches capable of increasing sensitivity and reducing analysis time, as reported by Rodrigues Spinelli et al. (2021). The authors claimed recovery values ranging from 90.7% to 108.6% introducing a pH adjustment, the use of cellulose ester membranes, precipitation with organic solvents, and a final concentration/clean-up. The LOQ values ranged from 0.01 to 0.25 μg/ml. Pig gelatin and egg white proteins have been determined in 5-year aged Nebbiolo-based red wine by Restani et al. (2014) and Dal Bello et al. (2021). Biomarker peptides were detected and quantified by a nano LC-HR-MS method.

Yang et al. (2021) have recently developed an LC-MS/MS method to quantify ovalbumin, ovotransferrin, β–lactoglobulin, and 4 caseins (α S1-, α S2-, β-, and κ–casein). The authors optimized the clean-up procedure for wine samples using an extraction treatment by a PVPP solution, trypsin digestion of proteins followed by peptide purification on HLB SPE cartridges to remove buffers, digest reagents, other matrix components, and interfering peptides. This article reported a standard addition strategy to quantify the wine allergens. The LODs for egg allergens were 0.1 μg/ml and for milk allergens 0.003–0.015 μg/ml, showing high sensitivity, simple clean-up procedure, and a reduction of costs for this approach.

Since MS approaches become increasingly customary, it has started to appear in literature articles dealing with the investigation of a large number of wine samples to detect and quantify exogenous protein residues. Jessy Pavón-Pérez et al. (2019) analyzed white and red wines from four different grape varieties produced by 14 Chilean wineries. This approach to determining casein and ovalbumin is based on ultrafiltration membranes, protein precipitation with organic solvents, followed by a fast (7 h) enzymatic digestion. The LOD and LOQ achieved using an LC-MS/MS approach ranged from 4.70 to 8.50 μg/L and 10 to 20 μg/L, respectively.

### Biosensors

Since the ELISA approach is somewhat doubtful as far as it concerns false negatives or positives (Lacorn et al., 2011), and the MS approach requires pricey instrumentation and more specialized operators, it was reasonable to expect the development of simpler and still reliable approaches to determine protein residues in wines. Up to date, several electrochemical biosensors have been proposed for the detection of food allergens (lysozyme, ovalbumin, caseins, and lactoglobulin) (Vasilescu et al., 2016), but only a few of them have been tested on wines (Ocaña et al., 2015; Mihai et al., 2015; Pilolli et al., 2015; Wessels and Paschke-Kratzin, 2016; Titoiu et al., 2019). The recent availability of several aptamer sequences, specifically synthesized for lysozyme capture, prompted the development of several sensors for the detection of lysozyme in wines (Mihai et al., 2015; Ocaña et al., 2015; Titoiu et al., 2019). Aptamers are short oligonucleotide or peptide sequences that are more stable with respect to antibodies, with the further advantage of reduced production costs. The LOD values for such biosensors range from few ppt to ppm levels (Vasilescu et al., 2016). On the other hand, Pilolli et al. (2015) and Pilolli and Monaci (2016) worked on the development of a surface plasmon resonance (SPR)-based biosensor to detect egg-related fining allergens. The same group refined the approach (2016) in order to increase the sensitivity while still maintaining the speed and easy sample handling of the sensor-based instrumentation. Indeed, wine samples were subjected to a very short pretreatment by PVP/SEC purification steps that allowed reaching a LOD of up to 0.2 μg/ml. Recently, Baldo et al. (2021) and Rodrigues Spinelli et al. (2021) described the combination of a disposable electrochemical device with magnetic beads, delivering an ultrasensitive detection of the egg allergen ovalbumin in wines. The approach leads to a LOD of 0.2 fg/ml within a wide linear range of concentration from 0.01 to 10 pg/ml (European Commission, 2003; European Commission, 2007).

## Conclusion

The determination of protein residues in wines remains a strong topic in the current research. While early applications relied on the more economical ELISA approach, in recent years, HPLC/MS has gained new ground due to a lower risk of false positives and substantial sensitivity improvements. These improvements, however, do not come cheap since the quantitative determination of protein residues in wines is complicated by several steps of sample treatment, analyte recoveries, and ionization efficiency of the peptides quantified. Thus, in order to reduce costs and improve determination time, recently, some sensor-based applications have started to appear. In wines, their application is actually limited to lysozyme and ovalbumin detection, but it is foreseeable that their wide-spread use will obtain reliable results at very reasonable costs since their development is rapidly gaining momentum.
